# NEAR-INFRARED SPECTROSCOPY IN VIVO DISTINGUISHES AMYLOID DEPOSITION IN SUBJECTS WITH ALZHEIMER’S DISEASE

**DOI:** 10.1093/geroni/igad104.3490

**Published:** 2023-12-21

**Authors:** Brent Schell, Frank Greco

**Affiliations:** Boston Medical Center/ Boston University School of Medicine, Boston, Massachusetts, United States; VA Bedford Healthcare System, Bedford, Massachusetts, United States

## Abstract

Cerebral amyloid angiopathy (CAA), a condition characterized by amyloid beta peptide deposition in cerebral vasculature, is commonly found in patients with Alzheimer’s disease (AD). Previous studies have shown that it is possible to distinguish subjects with Alzheimer’s disease from age-matched controls in vivo using near-infrared (NIR) spectroscopy. However, correlating neuropathological findings with the spectral features that best distinguish disease from control remains a challenge. Tissue optics predict that pathology in the leptomeningeal vasculature should be distinguishable from that of the adjacent brain parenchyma. In this study, NIR spectra were acquired in vivo from subjects with autopsy confirmed AD (n= 26). Postmortem specimens were histologically classified according to the degree of CAA (0 to 3+) noted in the leptomeningeal vasculature for each patient. A feature selection approach was used to discover spectral features that distinguished patients with high versus minimal degrees of CAA found at autopsy (scored as 3+ vs. 0, respectively). Two distinct spectral regions around 768nm and 891nm were identified as discriminants which could properly classify patients according to their CAA score, suggesting that these regions are correlated with amyloid beta peptide deposition. These results suggest that NIR spectroscopy can be used to detect and possibly inform the burden of amyloid deposition. More research is needed to establish if this technique can be used to monitor disease progression and response to therapeutic interventions, especially in light of emerging new immunotherapies.

